# Functional Epigenetic Analysis of Prostate Carcinoma: A Role for Seryl-tRNA Synthetase?

**DOI:** 10.1155/2014/362164

**Published:** 2014-03-27

**Authors:** Odiljon Ikromov, Imad Alkamal, Ahmed Magheli, Nadine Ratert, Mauricio Sendeski, Kurt Miller, Hans Krause, Carsten Kempkensteffen

**Affiliations:** ^1^Klinik für Urologie, Charité-Universitätsmedizin Berlin, Charité Campus Mitte (CCM), Charitéplatz 1, 10117 Berlin, Germany; ^2^Institut für Vegetative Physiologie, Charité-Universitätsmedizin Berlin, Charitéplatz 1, 10117 Berlin, Germany

## Abstract

Transcriptional silencing, as a result of aberrant promoter hypermethylation, is a common mechanism through which genes in cancer cells become inactive. Functional epigenetic screens using demethylating agents to reexpress transcriptional silenced genes may identify such inactivated genes for needing further evaluation. We aimed to identify genes so far not known to be inactivated by promoter hypermethylation in prostate cancer. DU-145 and LNCaP cells were treated with the DNMT inhibitor zebularine. Expression changes of total RNA from treated and untreated cells were compared using an RNA expression microarray. Genes upregulated more than 2-fold were evaluated by RT-qPCR in 50 cases of paired normal and tumor tissues of prostate cancer patients. SARS was found to be downregulated in prostate cancer in 42/50 cases (84%). In addition, GADD45A and SPRY4 showed a remarkable diminished expression (88% and 74%, resp.). The gold standard for promoter hypermethylation-inactivated genes in prostate cancer (GSTP1) was repressed in 90% of our patient samples. ROC analyses reported statistically significant AUC curves in SARS, GADD45A, and GSTP1 and positive Spearman correlations were found between these genes. SARS was discovered to be a novel gene that is repressed in prostate cancer and could therefore be recommended for its involvement in prostate carcinogenesis.

## 1. Introduction

Prostate cancer (PCa) is the second most frequently diagnosed cancer and the fifth leading cause of cancer death among men worldwide, with 1111,689 new cases and 307,471 (6.6%) deaths projected to occur in 2012 [1]. In 2013, cancers of the prostate, lung and bronchus, and colorectum will account for about 50% of all newly diagnosed cancers in the United States; prostate cancer alone will account for 28% (238,500) of incident cases [2]. In Germany, prostate cancer is already the third most cause of cancer death with more than 68,000 newly diagnosed cases (25.2%) and a mortality rate of approximately 12,500 men (10.7%) in 2012 [1]. Despite extensive scientific efforts and technological progress, the molecular mechanisms of development and progression in particular to lethal PCa are still elusive and need further investigation.

Genetic and epigenetic alterations in prostate tumorigenesis, as in all other cancers, accumulate within a multistep transformation process on the cellular level [3, 4]. Aberrant DNA promoter CpG islands hypermethylation of genes is a well-characterized mechanism for transcriptional silencing of tumour suppressor genes in various cancers [5]. Epigenetic silencing associated with promoter hypermethylation has been well documented also in PCa, including GSTP1, RARB, APC, PYCARD, PTGS2, ABCB1, and RASSF1 genes [6].

One way to identify epigenetically silenced genes in tumor cells is based on reversal of epigenetic silencing by DNA methyltransferases (DNMT) inhibitors such as 5-aza-2-deoxycytidine and the structurally related compound zebularine [7]. We used moderate concentrations of zebularine to reactivate epigenetically silenced genes by means of demethylating cytosine residues resulting in the “reexpression” of the respective genes [8]. Compared to other more frequently used drugs such as azacytidine and decitabine, zebularine is a highly stable hydrophilic inhibitor of DNA methylation with oral bioavailability and low toxicity even after prolonged administration [9, 10]. We treated androgen insensitive DU-145 and androgen sensitive LNCaP PCa cell lines with moderate doses of zebularine and identified globally silenced genes by RNA microarray analysis of the transcriptome. Through intuitive selection of upregulated and validated genes, we estimated relative gene expression (RGE) profiles for selected candidate genes in 50 patients using paired samples of adjacent normal and tumour prostate tissue. In agreement with our working hypothesis of methylation-induced transcriptional inactivation, we focused our attempts on genes that are exclusively downregulated in primary PCa. Eventually, we identified, for the first time, seryl-tRNA synthetase (SARS) to be downregulated in almost all PCa cases analysed. SARS was further characterized to qualify as a possible candidate for future epigenetic study approaches in PCa.

## 2. Materials and Methods

### 2.1. Zebularine Treatment

LNCaP prostate cancer cell lines (ATCC number: CRL-1740) and DU-145 (ATCC number: HTB-81) were chosen for treatment experiments. Both cell lines were purchased from the American Type Culture Collection (ATCC, Manassas, VA, USA). Cells were treated with a final concentration of 100 *μ*M of the DNA methyltransferase (DNMT) inhibiting reagent zebularine. Growth medium containing zebularine was replaced regularly after 48–72 hours and cells were split at a ratio of 1 : 3. In total, cells were exposed to zebularine for 216 hours. Three independent treatment experiments including untreated controls were performed.

### 2.2. Tissue Samples

Tumor and adjacent normal tissues samples of prostate cancer patients collected after radical prostatectomy between 2002 and 2004 at the Department of Urology, Charité-Universitätsmedizin Berlin, Germany, were used in experiments. The samples were collected immediately after surgery in liquid nitrogen and stored at −80°C. The study was done according to the regulation of ethical board of the hospital with informed consent of patients. Samples were analyzed by a uropathologist for their tumor content. In total, 50 prostate adjacent normal and tumor tissue samples containing at least 60% tumor tissue with different stage were included in this study. Patients' clinical parameters are described in Table 1.

### 2.3. RNA Isolation and cDNA Synthesis

Total RNA was extracted from treated and untreated PCa cells as well as from prostate normal and tumor tissue samples by using the “miRNeasy Mini Kit” (Qiagen, Hilden, Germany) according to manufacturer's recommendations. Approximately, 4-5 × 10^6^ cells and 20–30 mg of tissue were used for RNA extraction. RNA concentration and purity were determined spectrophotometrically on a Nanodrop ND-1000 instrument (Thermo Scientific, Germany). All RNA samples were free from remaining proteins (260/280 nm ratio from ~1.8 to 2.0) and other contaminations (260/230 nm ratio = 2.0 to 2.2). In addition, integrity of RNA was assessed by capillary electrophoresis on the Bioanalyzer-2100 instrument (Agilent Technologies, Waldbronn, Germany). Only cell line RNA samples with RIN numbers ≥8.0 were used for RNA chip analysis. For RT-qPCR analyses, only tissue RNAs with RIN ≥6.0 were considered. Subsequently, RNA was reverse transcribed according to the manufacturer's instructions of the “Transcriptor First Strand cDNA Synthesis Kit” (Roche Diagnostics, Mannheim, Germany). To increase sensitivity, a combination of anchored-oligo (dT) priming and random hexamer priming was used to transcribe 1 *μ*g RNA in a total volume of 10 *μ*L (Supplementary File S1) (see Supplementary Material available online at http://dx.doi.org/10.1155/2014/362164).

### 2.4. Microarray Analyses

RNA microchip analyses were performed at the core facility “Labor für funktionelle Genomforschung” (LFGC) of Charité-Universitätsmedizin Berlin. Totally, 12 Affymetrix 1.0 ST chip (Cat. number 901086, Affymetrix Inc., Santa Clara, CA, USA) hybridization analyses for both PCa treated and untreated cells lines were performed in triplicate. This type of chip covers 36079 probes that represent 21014 genes. 300 ng of total RNA was used for first cycle cDNA synthesis from both treated and untreated PCa cells. Labeled cDNA was hybridized at 45°C for 16 hrs. Staining and washing were performed in a Fluidics Station 450 (Affymetrix Inc., Santa Clara, CA, USA). The scanning was carried out on a GeneChip Scanner 3000 G7 system (Affymetrix Inc., Santa Clara, CA, USA). The raw data were normalized according to the log scale robust multiarray analysis (RMA) [11]. Briefly, signal intensities were background adjusted to the perfect match (PM) intensities and quantile normalization approach was performed across all arrays of the experiment. After log2, transformation data were global median polished. In order to control the false discovery rate at *α* < 0.05 for array data, we applied the false discovery rate multiple testing correction, according to Benjamini and Hochberg [12]. All RNA chip data have been deposited in the National Center for Biotechnology Information GEO database under accession number GSE51629 (http://www.ncbi.nlm.nih.gov/geo/query/acc.cgi?acc=GSE51629).

### 2.5. Computational Analyses

Differentially expressed genes were identified using Microarray Suit 4.0 software (Affymetrix Inc., Santa Clara, CA, USA). Candidate genes were selected according to their fold change upregulation with a cutoff of 2-fold. In addition, we applied the presence of one or more CpG islands in the promoter region of a particular gene as second criteria. EMBOSS CpGPlot program (http://www.ebi.ac.uk/Tools/seqstats/emboss_cpgplot/) was used to determine the presence of CpG islands of the respective genes. Furthermore, serial analysis of gene expression (SAGE, http://cgap.nci.nih.gov/SAGE/) was exploited to check for candidates that show the same or even higher expression in normal tissue when compared to tumor tissue.

### 2.6. Quantitative Real-Time PCR (RT-qPCR)

RT-qPCR for selected genes was performed on the LightCycler 480 Instrument (Roche Diagnostics GmbH, Mannheim, Germany) in 96-well white plate format and analyzed using proprietary software (v.1.5.0). RT-qPCR was carried out according to the manufacturer's protocol and the MIQE guidelines (Supplementary File S2). For relative quantification, 1/10 of cDNA was amplified using the “Probe Master kit” and proprietary “UPL probe” PCR format (Roche) in a total reaction volume of 10 *μ*L. Primer sequences and appropriate probe sets for target and reference genes were derived from the UPL website (http://www.roche-applied-science.com/sis/rtpcr/upl/ezhome.html). Amplification primers were synthesized by TIB MOLBIOL (TIB MOLBIOL, Berlin, Germany); UPL hydrolysis-type probes were purchased from Roche Diagnostics GmbH. PCR was run with a preincubation at 95°C for 10 minutes, followed by cycling (45x) at 95°C/10 sec and 59°C/20 sec (Supplementary File S1). All samples were measured in triplicate; each PCR run includes no-template control and interpolate calibrator. mRNA expression levels were normalized for intra- and interassay variation by inclusion of a calibrator and PBGD as the reference gene. PCR efficiency was determined using standard curves and ranged between 90% and 98% (Supplementary File S3). Data were analyzed using GenEx Software v. 4.3.7 (MultiD Analyses AB, Göteborg, Sweden, http://multid.se/).

### 2.7. Data Analyses

Statistical analyses of gene expression data were performed using the statistical programs GraphPad Prism v.6.01 (GraphPad Software Inc., La Jolla, CA) and SPSS 19.0 (IBM Corporation, Somers, NY). Significant differences between paired normal and tumor tissues were calculated using nonparametric Wilcoxon test. Spearman's rank correlations were used to analyze the relationships between expression levels of deregulated genes, as well as between clinical variables. To determine the discriminative potential of deregulated genes between normal and tumor samples and the diagnostic accuracy, we used receiver operating curves (ROC) calculated by MedCalc Software v.12.0 (MedCalc Software, Mariakerke, Belgium). Sample size calculation (*α* = 5%; power = 80%) for the comparison of the area under the receiver operating characteristics curve of 0.8 (taking into account this value as appropriate discrimination power) with the null hypothesis value 0.5 was calculated to be 28 in each group. *P* values <0.05 (two-tailed) were considered statistically significant in all cases.

## 3. Results and Discussion

Epigenetic screens exploiting the effects of demethylating agents to reactivate transcriptionally silenced genes are suitable tools for the discovery of new biomarkers and putative therapeutic targets [13].

**Figure 1:**
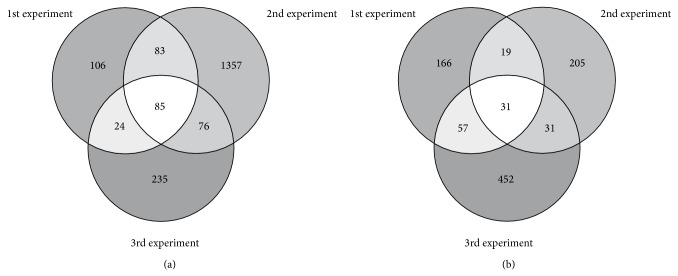
Number of shared ≥1.5-fold upregulated genes in Venn diagrams in three independent biological experiments in PCa cell lines DU-145 (a) and LNCaP (b) after treatment with the demethylating agent zebularine.

We performed an RNA microarray screen of zebularine-treated and zebularine-untreated prostate cancer cells lines DU-145 and LNCaP and discovered a total of 3449 genes expressed at least ≥1.5-fold in 3 independent experiments. The number of shared upregulated genes in both cell lines was 85 and 31, respectively (Figure 1).

A total of 91 genes were at least 2-fold upregulated in the two prostate cancer cell lines. By applying additional selection criteria, like the presence of CpG islands in the promoter region and SAGE database-derived expression data towards an elevated intrinsic expression in normal prostate tissues, the number of candidates was further reduced to 52 (Table 2).

Particular emphasis was added on the efficacy of zebularine action, since this drug is known to be less active when compared to other demethylating agents. Therefore, we measured the “demethylating potential” of zebularine indirectly by measuring the reexpression of IFI6 gene that is regulated by hypermethylation of its promoter [14]. Using RT-qPCR, we measured a 19.4-fold (SD ± 4.6) upregulation of IFI6 after treatment with 100 *μ*M zebularine for 9 days.

**Figure 2:**
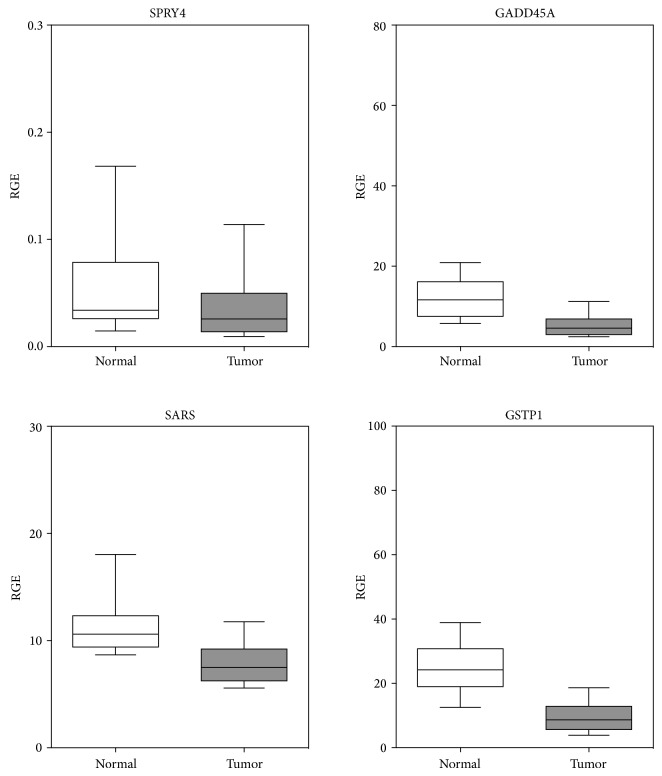
Expression of downregulated candidate genes SPRY4, SARS, GADD45A, and GSTP1 in prostate nonmalignant and malignant tissue samples. RT-qPCR was performed from 50 paired prostate tissue samples. Values are given in boxes (white: nonmalignant; black: malignant) that represent lower and upper quartiles with medians as horizontal line. Whiskers depict the 10–90 percentiles. Considered significances (*P* < 0.05) are calculated with Wilcoxon signed rank test for all genes. Data are given in Table 3.

Next, we aimed to validate our candidates in clinical samples to provide evidence that they are indeed diminished in their expression in prostate carcinoma tissues. For verification of our screening assay, we were looking specifically for sprouty homolog 4 (SPRY4) and growth arrest and DNA-damage-inducible, alpha (GADD45A), since these genes were already revealed by similar epigenetic reactivation screens [15–17]. Central to our measures of relative gene expression (RGE) in 50 matched tumor and normal prostate tissues, however, was the gene for seryl-tRNA synthetase (SARS), so far not described for its diminished expression in prostate cancer. As a gold standard for subsequent comparisons, we used the frequently suppressed glutathione S-transferase *π*1 (GSTP1, Figure 2) [18] that was downregulated in 45 of our prostate tumor tissues (90%, *P* < 0.0001) with median fold change of −2.54 (Table 3).

SPRY4 was significantly downregulated in this study in 37 tumor specimens (74%, *P* = 0.0007, Figure 2) and most of the samples (31/37; 84%) were inhibited more than 1.5-fold (median = −1.64, Table 3). Wang et al. reported that the expression of this inhibitor of the growth factor-induced cell responses was downregulated in approximately half of prostate cancers due to promoter hypermethylation. This regulation of SPRY4 by epigenetic inactivation was further substantiated using 5 Aza-dC treatment of LNCaP cells that restored its expression [16]. These results and the observation that the close relative SPRY2 is also regulated by epigenetic modification add further evidence on the role of members of the SPRY family as putative tumor suppressors in prostate cancer [19].

Another gene that is repressed by promoter hypermethylation is the cell cycle regulator GADD45A (also known as DDIT1) that causes cell cycle arrest by blocking G2-M transition in response to cellular DNA damage [20]. In agreement with findings by Lodygin et al. [15], we found that this gene repressed in 44 tumor tissues (88%, *P* < 0.0001) with a majority of samples downregulated more than 1.5-fold (median = −2.32, Table 3 and Figure 2). The role of GADD45A as an epigenetically regulated and putative therapeutic target is also emphasized by the observation that DNMT inhibitors enhance sensitivity to docetaxel in DU-145 and LNCaP cell lines [21].

Surprisingly, expression of seryl-tRNA synthetase (SARS, SERS, or SERRS) was significantly repressed in the majority of our prostate cancer specimens, although we are aware of evidences for aminoacyl-tRNA synthetases in cancer progression [22]. SARS expression was significantly diminished in 42 cases (84%, *P* < 0.0001) when compared to matched normal tissue and more than half of the downregulated samples (23/42; 55%) were repressed more than 1.5-fold (median = −1.44) (Table 3 and Figure 2). With regard to SARSs possible involvement in prostate carcinogenesis, recently, a unique domain at the C-terminus of almost all vertebrate SARSs (named UNE-S) that links SARS to vascular development by its interaction with vascular endothelial growth factor A (VEGFA) was described [23]. VEGFA itself is a key regulator of angiogenesis that activates tyrosine kinase receptors VEGFR1 (Flt-1) and VEGFR2 (KDR/Flk-1), respectively [24]. Our own group recently aimed to link angiogenesis-related factors more closely to prostate cancer progression by discovering decreased transcript levels of VEGFR2 and other endothelial factors such as CD34, CD146, and CAV1 in prostate cancer [25]. It is also noteworthy that SARS is an essential regulatory component of selenium metabolism, whereby dietary selenium levels themselves are still a controversial issue in prostate cancer chemoprevention [26, 27].

**Figure 3:**
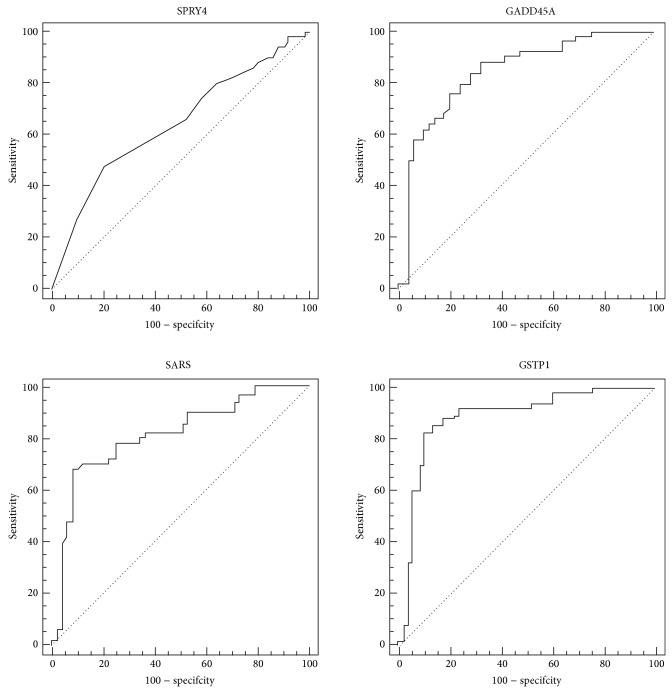
Receiver operating characteristic (ROC) curve for the significantly downregulated candidate genes GADD45A, SARS, SPRY4, and GSTP1 to discriminate between tumor and adjacent normal samples. Comparison of area under the receiver operating characteristic curve (AUC) of candidate genes with AUC of GSTP1. Data are given in Table 4.

Next, we performed receiver operating characteristic (ROC) curve analyses of SARS RGE data and compared them to SPRY4, GADD45A, and the reference GSTP1 (Table 4). ROC area-under-curve (AUC) for SARS (0.816), GADD45A (0.841), and GSTP1 (0.884) was almost the same with regard to value and shape (Table 4 and Figure 3), The AUC of SPRY4 compared less well to the other genes (AUC = 0.644). In addition, it should be noted that, at a sensitivity cutoff of 90%, SARS showed only moderate specificity (48%).

Spearman correlation of ration of SARS expression data with proven epigenetically regulated GADD45A and GSTP1 resulted in a positive correlation among these genes (Table 5). However, we did not find any significant correlation of expression data with pathological parameters like tumor stage, grade, and Gleason scores (data not shown). Since methylation events are known to be differentiation- and age-dependent and occur early in carcinogenesis [28], we specifically tried to correlate SARS expression to the age of our patients. A Spearman correlation coefficient of *r* = −0.1626  (*P* = 0.2591) does not demonstrate any significant relationships.

## 4. Conclusion

An RNA microarray screen for epigenetically silenced genes in two prostate cancer cell lines revealed 52 candidates that were further analyzed in patient samples for their involvement in cancer development. We were able to verify, in the majority of our samples (88 and 74%), a diminished expression of GADD45 and SPRY4 that were known to be inactivated by hypermethylation in prostate cancer. Comparable results were obtained for a hitherto unknown transcript that now might be linked to prostate carcinogenesis, too. The enzyme SARS that interacts with components of VEGF and selenium pathways was found consistently downregulated in more than 80% of tumor tissues. Therefore, we believe that SARS might be a promising putative target for further (epi)genetic studies in prostate cancer.

## Supplementary Material

Supplementary File S1: comprise detailed information on cDNA synthesis and RT-qPCR
Supplementary File S2: Description of the experimental details of the RT-qPCR analyses according to the checklist of the MIQE guidelines
Supplementary File S3: qPCR validation experiments according to the MIQE guidelines with respect to the calibration curves and the dynamic range of measurements

## Figures and Tables

**Table 1:** Patients' clinical parameters.

Characteristics	Parameters	Patients *n* = 50 (100%)
Age, years	Median	64
	Range	47–74

Preoperative PSA, ng/mL	Median	8.7
	Range	1.06–78

T stage	pT2a	1 (2)
	pT2b	15 (30)
	pT2c	12 (24)
	pT3a	6 (12)
	pT3b	13 (26)
	pT3x	1 (2)
	pT4	2 (4)

N stage	N0/Nx	44 (88)
	N1	6 (12)

M stage	M0	50 (100)

Gleason score	n/a	1 (2)
	3	1 (2)
	5	15 (30)
	6	12 (24)
	7a (3 + 4)	9 (18)
	7b (4 + 3)	4 (8)
	8	4 (8)
	9	4 (8)

**Table 2:** List of upregulated genes after bioinformatics analyses^1^.

Gene symbol^2^	Gene name	Location	Fold change	CpG island^3^	Digital Northern^4^
ADRA2A	adrenergic, alpha-2A-, receptor	10q25.2	2.31	YES	D
DDX60	DEAD (Asp-Glu-Ala-Asp) box polypeptide 60	4q32.3	2.97	yes	S
**POTEF**	POTE ankyrin domain family, member F	2q21.1	2.15	yes	D
DDX58	DEAD (Asp-Glu-Ala-Asp) box polypeptide 58	9p12	3.75	yes	S
IGF1R	Insulin-like growth factor 1 receptor	15q26.3	2.72	yes	S
INPP4B	Inositol polyphosphate-4-phosphatase, type II	4q31.21	2.63	yes	D
**IFI6**	Interferon, alpha-inducible protein 6	1p35	4.86	yes	D
*TXNIP* ∗	Thioredoxin interacting protein	1q21.1	4.65	YES	D
ADAM32	ADAM metallopeptidase domain 32	8p11.22	3.73	yes	S
STC2	Stanniocalcin 2	5q35.1	3.68	YES	S
BEST1	Bestrophin 1	11q13	3.63	YES	S
**ASNS**	Asparagine synthetase (glutamine-hydrolyzing)	7q21.3	3.39	YES	D
**CTH**	Cystathionase (cystathionine gamma-lyase)	1p31.1	3.36	yes	S
C12orf39	Chromosome 12 open reading frame 39	12p12.1	3.19	YES	S
JHDM1D	Jumonji C domain containing histone demethylase 1 homolog D (*S. cerevisiae*)	7q34	3.08	yes	S
PPP1R15A	Protein phosphatase 1, regulatory subunit 15A	19q13.2	2.99	YES	S
***SPRY4*** ∗	Sprouty homolog 4 (*Drosophila*)	5q31.3	2.96	YES	S
ZC3H6	Zinc finger CCCH-type containing 6	2q13	2.87	yes	S
TMEM156	Transmembrane protein 156	4p14	2.87	yes	S
FAM129A	Family with sequence similarity 129, member A	1q25	2.80	yes	S
CDRT1	CMT1A duplicated region transcript 1	17p12	2.74	yes	S
UPP1	Uridine phosphorylase 1	7p12.3	2.71	yes	D
MAP2	Microtubule-associated protein 2	2q34-q35	2.65	yes	S
MOCOS	Molybdenum cofactor sulfurase	18q12	2.59	yes	S
C6orf48	Chromosome 6 open reading frame 48	6p21.3	2.58	YES	S
PYROXD1	Pyridine nucleotide-disulphide oxidoreductase domain 1	12p12.1	2.54	yes	S
ZNF814	Zinc finger protein 814	19q13.43	2.52	yes	S
CLDN1	Claudin 1	3q28-q29	2.52	yes	S
**ABLIM3**	Actin binding LIM protein family, member 3	5q32	2.52	yes	D
DDIT4	DNA-damage-inducible transcript 4	10q22.1	2.51	YES	S
TFPI2	Tissue factor pathway inhibitor 2	7q22	2.51	YES	S
TUBE1	Tubulin, epsilon 1	6q21	2.45	yes	D
***GADD45A*** ∗	Growth arrest and DNA-damage-inducible, alpha	1p31.2	2.36	YES	S
FRZB	Frizzled-related protein	2qter	2.34	yes	S
C5orf28	Chromosome 5 open reading frame 28	5p12	2.33	yes	D
SERPINB8	Serpin peptidase inhibitor, clade B (ovalbumin), member 8	18q22.1	2.33	yes	S
ZNF300	Zinc finger protein 300	5q33.1	2.32	yes	S
ZDHHC11	Zinc finger, DHHC-type containing 11	5p15.33	2.29	yes	S
GTPBP2	GTP binding protein 2	6p21	2.27	YES	D
MKX	Mohawk homeobox	10p12.1	2.24	yes	S
CD274	CD274 molecule	9p24	2.22	yes	S
ZNF643	Zinc finger protein 643	1p34.2	2.19	yes	S
C9orf150	Leucine rich adaptor protein 1-like	9p23	2.19	yes	S
TES	Testis derived transcript (3 LIM domains)	7q31.2	2.17	yes	S
PSAT1	Phosphoserine aminotransferase 1	9q21.2	2.16	YES	S
PAX6	Paired box 6	11p13	2.15	YES	S
ETV5	ETS variant 5	3q28	2.11	yes	S
**SARS**	Seryl-tRNA synthetase	1p13.3	2.10	YES	D
CD226	CD226 molecule	18q22.3	2.04	yes	S
GDPD1	Glycerophosphodiester phosphodiesterase domain containing 1	17q22	2.01	yes	S
LETM2	Leucine zipper-EF-hand containing transmembrane protein 2	8p11.2 3	2.01	YES	S

^1^ Selected 52 genes that showed at least ≥2-fold upregulation in two prostate cancer cell lines.

^2^Gene names in bold were analyzed by RT-qPCR. Gene name in italic with asterisk indicates gene previously identified and hypermethylated in PCa but not analyzed by us. Gene names in bold italic with asterisk indicate genes previously identified and hypermethylated in PCa and analyzed in this paper.

^3^Various writing styles indicate the size of CpG island(s): YES, the largest size; yes, the smallest size.

^4^S: equally expressed in normal and malignant tissue; D: increased expression in normal prostate tissue.

**Table 3:** mRNA expression changes of candidate genes represented by statistical analysis∗.

Genes	*P* value	Downregulation prevalence (normal versus tumor)	Fold changes (normal versus tumor)	≥−1.5-fold changes prevalence (normal versus tumor)
SPRY4	0.0007	74% (37/50)	−1.64	84% (31/37)
SARS	<0.0001	84% (42/50)	−1.44	55% (23/42)
GADD45A	<0.0001	88% (44/50)	−2.32	84% (37/44)
GSTP1	<0.0001	90% (45/50)	−2.54	84% (42/45)

^*^Considered significances (*P* < 0.05) calculated with Wilcoxon signed rank test for all genes. Normal versus tumor common downregulation and ≥−1.5-fold changes prevalence among downregulated samples.

**Table 4:** Performance of significantly downregulated candidate genes to discriminate between malignant and nonmalignant samples∗.

Gene	Sensitivity (95% CI)	Specificity (95% CI)	AUC	*P* value	Standard error
GSTP1	90% (78.2–96.7)	78% (64.0–88.5)	0.884	<0.0001	0.0369
GADD45A	90% (78.2–96.7)	58% (43.2–71.8)	0.841	<0.0001	0.0407
SARS	90% (78.2–96.7)	48% (33.7–62.6)	0.816	<0.0001	0.044
SPRY4	90% (78.2–96.7)	16% (7.2–29.1)	0.644	0.0085	0.0549

^*^Five candidate genes that show significant downregulation on Wilcoxon signed rank test and further performance assessed by ROC curve analysis. 90% sensitivity and specificity with 95% confidence interval (95% CI).

**Table 5:** Spearman rank correlation coefficients by ration of expression between downregulated candidate genes.

Factor∗	SPRY4	SARS	GADD45A	GSTP1
SPRY4	—	0.236	0.428^b^	0.347^a^
SARS	0.236	—	0.591^c^	0.685^c^
GADD45A	0.428^b^	0.591^c^	—	0.644^c^
GSTP1	0.347^a^	0.685^c^	0.644^c^	—

^*^Significantly downregulated candidate genes. Correlation coefficient (*r*_*s*_) values and *P* values are shown: ^a^ *P* < 0.05; ^b^ *P* < 0.01; ^c^ *P* < 0.001.
