# Supplementary material for: Functional Epigenetic Analysis of Prostate Carcinoma: A Role for Seryl-tRNA Synthetase?
Source: J Biomark. 2014 Mar 27;2014:362164. doi: 10.1155/2014/362164 (PMC4437382; doi:10.1155/2014/362164)
Supplement: Supplementary file 1 — Supplementary File S1: comprise detailed information on cDNA synthesis and RT-qPCR Supplementary File S2: Description of the experimental details of the RT-qPCR analyses according to the checklist of the MIQE guidelines Supplementary File S3: qPCR validation experiments according to the MIQE guidelines with respect to the calibration curves and the dynamic range of measurements [file 362164.f1.pdf]

Supplementary file S1

cDNA synthesis

Table 1. Protocol of cDNA synthesis procedure.

"Transcriptor First-Strand cDNA Synthesis Kit" Roche Diagnostics (Mannheim, Germany) (Cat. No.04896866001).

Components	Volume and concentration
Total RNA	10μL (1μg)
Anchored-oligo (dT) primer	1μL (2.5μM)
Random hexamer primer	2μL (60μM)
**cDNA synthesis mix**	**Volume and concentration**
Transcriptor Reverse Transcriptase Reaction Buffer	4μL (1×) (8mM MgCl2)
Protector RNase Inhibitor	0.5μL (20U)
Deoxynucleotide Mix,10 mM	2μL (1mM)
Transcriptor Reverse Transcriptase	0.5μL (10U)

Table 2. Cycling protocol for reverse transcription of mRNA.

	Preparation	
Steps	Time	Temperature
Denaturation	15 min	65°C
	∞	4°C
	cDNA synthesis	
Annealing	10 min	25°C
Elongation	30 min	55°C
Inactivation	5 min	85°C
	∞	4°C

Quantitative real-time PCR (RT-qPCR).

Table 3. Primers and UPL probe for target gene.

Gene name/ accession number	Forward primers	Reverse primers	UPL probe	Amplicon (nt)
GADD45A NM_001924.3 variant 1	TTTGCAATATGACTTTGGAGGA	CATCCCCCACCTTATCCAT	19	72
SARS NM_006513.2	TGGGCAAACCAAGAAGATG	GCAGATGGTACGGGTAGTGG	39	85
IFI6	CTGTGCCCATCTATCAGCAG	GGGCTCCGTCACTAGACCTT	41	75

NM_022873.2<br>variant 3				
SPRY4<br>NM_030964.3<br>variant 1	CCCCGGCTTCAGGATTTA	CTGCAAACCGCTCAATACAG	17	85
GSTP1<br>NM_000852.3	TCTCCCTCATCTACACCAACTATG	AGGTCTTGCCTCCCTGGT	56	114

Table 4. Protocol of reaction mix using cDNAs and LightCycler480 Probes Master kit.

Probe Master kit (Cat. No.04707494001) and UPL probe from Roche Diagnostics GmbH (Mannheim, Germany). White 96-well PCR-plates (Cat. No.04729692001). Amplification primers for candidate genes were purchased from TIB MOLBIOL Berlin.

	Volume
H_20	3.4µL
Upstream-Primer (10µM)	0.25µL
Downstream-Primer (10µM)	0.25µL
Probe (Universal Probe Library)	0.1µL
Master Mix (2 x conc.)[1]	5µL
cDNA template	1µL
Total volume	10µl

2× conc., ready-to-use hot-start PCR mix, contains FastStart Taq DNA Polymerase, reaction buffer, dNTP mix (with dUTP instead of dTTP), and 6.4 mM $MgCl_2$.

Table 5. Cycling protocol for relative quantification on LC480.

Program	Temperature	Hold
Pre-incubation (1 cycle)	95°C	10:00 min
	95°C	00:10 sec
Amplification (45 cycles)	59°C	00:20 sec
	72°C	00:01 sec
Cooling (1 cycle)	40°C	00:30 sec

* For each cycle Mono-color FAM fluorescence measurements with wave length of 483-533nm was used.

The analytical precision of the qPCRs (the standard deviation of the Ct values) was tested by intra-run (n=10) measurements for gene FABP6. Ct value ranged between 22.65 and 22.78, median-22.67, SD±0.044, SD% 0.19.

Reference genes.

Commercially available mRNA-specific Roche Diagnostics GmbH Ready-to-Use reference genes assays were used to detect of expression of reference gene PBGD (Cat.No.05046149001), HPRT1 (Cat.No.05046157001) and TBP (Cat.No.05189284001). Further analyses for assessing their contribution as normalizer and compared suitability of housekeeping genes were done by geNorm^{PLUS} software (Biogazelle, Belgium). In our case gene with the least expression level was TBP (M=0.6850) and the most stable one was PBGD (M=0.5950). Average stability level showed gene HPRT1 with M value 0.6225.
