# Supplementary material for: Functional Epigenetic Analysis of Prostate Carcinoma: A Role for Seryl-tRNA Synthetase?
Source: J Biomark. 2014 Mar 27;2014:362164. doi: 10.1155/2014/362164 (PMC4437382; doi:10.1155/2014/362164)
Supplement: Supplementary file 2 [file 362164.f2.pdf]

Supplementary file S2.

Description of the experimental details of the RT-qPCR analyses according to the checklist of the MIQE guidelines.

The items in the checklist summarize the characteristics of the RT-qPCR analyses according to Bustin et al. [Clin Chem (2009) 611-622] and indicate where the details are described in the manuscript.

ITEM TO CHECK	IMPOR-TANCE	CHECK-LIST	WHERE IN THE MANUSCRIPT; ADDITIONAL COMMENT
**ERXPERIMENTAL DESIGN**			
Definition of experimental and control groups	E	Yes	Materials and Methods: Patients and tissue samples.
Number within each group	E	Yes	Materials and methods: Patients and tissue samples. See also: Number and concordance of biological replicates.
Assay carried out by core lab or investigator´s lab?	D	Yes	All assays were performed in investigator´s lab.
Acknowledgement of authors´ contributions	D	Yes	Contributors who do not meet the authorship as defined by the journal are listed in the Acknowledgement section.
**SAMPLE**			
Description	E	Yes	Materials and Methods.
Volume/mass of sample processed	D	Yes	Materials and Methods: RNA isolation and cDNA synthesis
Microdissection or macrodissection	E	Yes	Materials and Methods: RNA isolation and cDNA synthesis; macrodissections with histological verification.
Processing procedure	E	Yes	Materials and Methods: Tissue samples.
If frozen – how and how quickly?	E	Yes	Materials and Methods: Tissue samples.
If fixed – with what, how quickly?	E	N/A	-
Samples storage conditions and duration (esp. for FFPE samples)	E	Yes	Materials and Methods: Tissue samples.
**NUCLEIC ACID EXTRACTION**			
Procedure and/or instrumentation	E	Yes	Materials and Methods: RNA isolation and cDNA synthesis. References are indicated.
Name of kit and details of any modifications	E	Yes	Materials and Methods: RNA isolation and cDNA synthesis. “miRNeasy Mini Kit” (Qiagen, Hilden, Germany) Cat. No.217004
Source of additional reagents used	E	No	-
Details of DNase or RNase treatment	E	Yes	Treatment was not used
Contamination assessment (DNA or RNA)	E	Yes	Materials and Methods: RNA isolation and cDNA synthesis
Nucleic acid quantification	E	Yes	Materials and Methods: RNA

			isolation and cDNA synthesis
Instrument and method	E	Yes	Materials and Methods: RNA isolation and cDNA synthesis
Purity (A260/A280)	D	Yes	Materials and Methods: RNA isolation and cDNA synthesis
Yield	D	Yes	Materials and Methods: RNA isolation and cDNA synthesis
RNA integrity method/instrument	E	Yes	Materials and Methods: RNA isolation and cDNA synthesis; Bioanalyzer 2100 (Agilent).
RIN/RQI or Cq of 3`and 5`transcripts	E	Yes	RIN values given in Materials and Methods: RNA isolation and cDNA synthesis.
Electrophresis traces	D	No	-
Inhibition testing (Cq dilutions, spike or other)	E	Yes	Dilution experiments were performed; PCR efficiencies were found 90%-98%; see also Supporting Information: Supplementary file S3. For all clinical samples, identical isolation procedures were performed.
REVERSE TRANSCRIPTION			
Complete reaction condition	E	Yes	Materials and Methods: Quantitative real-time PCR. Supporting Information Supplementary file S1; table 1.
Amount of RNA and reaction volume	E	Yes	Materials and Methods: Quantitative real-time PCR. Supporting Information: Supplementary file S1; table 1.
Priming oligonucleotide (if using GSP) and concentration	E	Yes	Materials and Methods: Quantitative real-time PCR. Supporting Information: Supplementary file S1; table 3.
Reverse transcriptase and concentration	E	Yes	Materials and Methods: Quantitative real-time PCR. Supporting Information: Supplementary file S1; table 1.
Temperature and time	E	Yes	Materials and Methods: Quantitative real-time PCR and reference indicated there. Supporting Information: Supplementary file S1; table 2.
Manufacturer and reagents and catalogue numbers	D	Yes	Materials and Methods: Quantitative real-time PCR. Supporting Information: Supplementary file S1; table 1.
Cqs with and without RT	D*	Yes	There were no Cqs < 40 in reactions without RT.
Storage conditions of cDNA	D	Yes	cDNA stored at -20°C.
qPCR TARGET INFORMATION			
If multiplex, efficiency and LOD of each assay	E	N/A	-
Sequence accession number	E	Yes	Supporting Information: Supplementary file S1; table 3.
Location of amplicon	D	N/A	Supporting Information: Supplementary file S1; specificity guaranteed by the manufacturer.
Amplicon length	E	N/A	Supporting Information:

			Supplementary file S1; specificity guaranteed by the manufacturer.
In silico specificity screen (BLAST, etc.)	E	N/A	Specificity guaranteed by the manufacturer.
Pseudogenes, retropseudogenes or other homologs?	D	N/A	Specificity guaranteed by the manufacturer.
Sequence alignment	D	N/A	-
Secondary structure analysis of amplicon	D	N/A	-
Location of each primer by exon or intron (if applicable)	E	Yes	Use of mRNA specific UPL assays; specificity guaranteed by the manufacturer.
What splice variants are targeted?	E	Yes	Supporting Information: Supplementary file S1; table 3.
**qPCR OLIGONUCLEOTIDES**			
Primer sequences	E	N/A	Supporting Information: Supplementary file S1; table 3.
RT Primer DB Identification Number	D	N/A	-
Probe sequences	D**	N/A	-
Location and identity of any modifications	E	Yes	Supporting Information: Supplementary file S3; table 3.
Manufacture of oligonucleotides	D	Yes	TIB MOLBIOL Syntheselabor GmbH.
Purification method	D	N/A	-
**qPCR PROTOCOL**			
Complete reaction conditions	E	Yes	Materials and Methods: Quantitative real-time PCR. Supporting Information: Supplementary file S1; table 4 and 5.
Reaction volume and amount of cDNA/DNA	E	Yes	Materials and Methods: Quantitative real-time PCR. Supporting Information: Supplementary file S1; table 4.
Primer, (probe), Mg++ and dNTP concentration	E	Yes	Materials and Methods: Quantitative real-time PCR. Supporting Information: Supplementary file S1; table 4.
Polymerase identity and concentration	E	Yes	Materials and Methods: Quantitative real-time PCR. Supporting Information: Supplementary file S1; table 4.
Buffer/kit identity and manufacture	E	No	Materials and Methods: Quantitative real-time PCR. Supporting Information: Supplementary file S1; table 4.
Exact chemical constitution of the buffer	D	Yes	The manufacturer does not provide this information.
Additives (SYBR Green I, DMSO, ect.)	E	Yes	Assays without additional additives.
Manufacturer of plates/tubes and catalog number	D	Yes	Materials and Methods: Quantitative real-time PCR; Supporting Information: Supplementary file S1; table 4.
Complete thermo cycling parameter	E	Yes	Materials and Methods: Quantitative real-time PCR. Supporting Information:

			Supplementary file S1; table 5.
Reaction setup (manual/robotic)	D	Yes	Manual setup.
Manufacturer of qPCR instruments	E	Yes	LightCycler 480; see also Materials and Methods: Quantitative real-time PCR.
**qPCR VALIDATION**			
Evidence of optimization (from gradients)	D	Yes	Kits from Roche Diagnostics GmbH. Optimization guaranteed by the manufacturer.
Specificity (gel, sequence, melt, or digest)	E	Yes	Specificity guaranteed by manufacturer.
SYBR Green I, Cq of the NTC	E	Not applicable	-
Calibration curves with slope and Y-intercept	E	Yes	Material and Methods: Quantitative real-time PCR; see also Supporting Information: Supplementary file S3.
PCR efficiency calculated from slope	E	Yes	Material and Methods: Quantitative real-time PCR; see also Supporting Information: Supplementary file S3.
Confidence interval PCR efficiency or standard error	D	Yes	Supporting Information: Supplementary file S3.
r2 of standard curve	E	No	Not provided by the LightCycler 480 software.
Linear dynamic range	E	Yes	Supporting Information: Supplementary file S3.
Cq variation at lowest concentration of the linear interval of calibration curves	E	Yes	Supporting Information: Supplementary file S3.
Confidence intervals throughout range	D	No	-
Evidence for limit of detection	E	Yes	Supporting Information: Supplementary file S3. Measurements of all were in linear dynamic range. Thus, it was not necessary to determine the LOD.
If multiplex, efficiency and LOD of each assay	E	N/A	-
**DATA ANALYSIS**			
qPCR analysis program (source, version)	E	Yes	Materials and Methods: Quantitative real-time PCR.
Cq method determination	E	Yes	Cq >37 was decided as limit.
Outlier identification and disposition	E	Yes	There were no outliers.
Results of NTCs	E	Yes	NTC did not result in any amplification.
Justification of number and choice of reference genes	E	Yes	PBGD was used as reference gene. Supporting Information, Supplementary file S1; Reference genes.
Description of normalization method	E	Yes	Materials and Methods: Data analysis; GenEx v. 4.3.7.
Number and concordance of biological replicates	D	Yes	Legend of figure 3: adjacent nonmalignant samples n = 50; tumor samples n = 50.
Number and stage (RT or qPCR) of technical replicates	E	Yes	Materials and Methods: Quantitative real-time PCR; triplicate measurements.
Repeatability (intra-assay	E	Yes	Materials and Methods:

variation, %CV)			Supplementary file S1; RT-qPCR.
Reproducibility (inter-assay variation, %CV)	D	N/A	-
Power analysis	D	No	-
Statistical methods for result significance	E	Yes	Materials and Methods: Data analysis. Results and Figure legends.
Software (source, version)	E	Yes	Materials and Methods: Data analysis.
Cq or raw data submission RDML	D	No	-

E, essential information that must be submitted with the manuscript; D, desirable information that should be submitted with the manuscript if available.
