# Supplementary material for: Functional Epigenetic Analysis of Prostate Carcinoma: A Role for Seryl-tRNA Synthetase?
Source: J Biomark. 2014 Mar 27;2014:362164. doi: 10.1155/2014/362164 (PMC4437382; doi:10.1155/2014/362164)

Supplementary file S3.

qPCR validation experiments according to the MIQE guidelines with respect to the calibration curves and the dynamic range of measurements.

Calibration curves were generated with serial dilutions of cDNA. The Ct values were calculated automatically by the LightCycler software, release 1.5.0 using the "second derivative maximum" cycle analysis method. The slopes, intercepts, and errors of the regression lines of the calibration curves from these dilution series and the PCR efficiencies ($E=10^{-1}$/slope) including the dynamic range and the Ct variation at the lower limit (the endpoint of the linear dynamic range) were calculated by the LightCycler 480 software 1.5.0. Validation of the qPCR and calibration curves of the GADD45A, SARS and PBGD are exemplarily shown as follows.

Gene	PCR Efficiency	Slope	Y-intercept	Error[1]	Linear dynamic range[2]	Ct variation at lowest limit[3]
GADD45A	1.84	-3.773	18.60	0.02	18.1-29.90	0.4
SARS	1.87	-3.66	16.71	0.006	21.76-29.20	0.14
PBGD	1.8	-3.869	16.98	0.01	21.45-33.54	0.13

[1]The error value is the mean squared error of the single data points fit to the regression line, according to the definition given in the handbook of the LightCycler software.

[2]The linear dynamic range represents the range of the Ct values between the highest and the lowest concentration of linear interval of the calibration curve.

[3]Ct variation given as SD at the endpoint of the linear dynamic range that corresponds to the lowest concentration in the linear interval of the calibration curve.

Standard curve for GADD45A

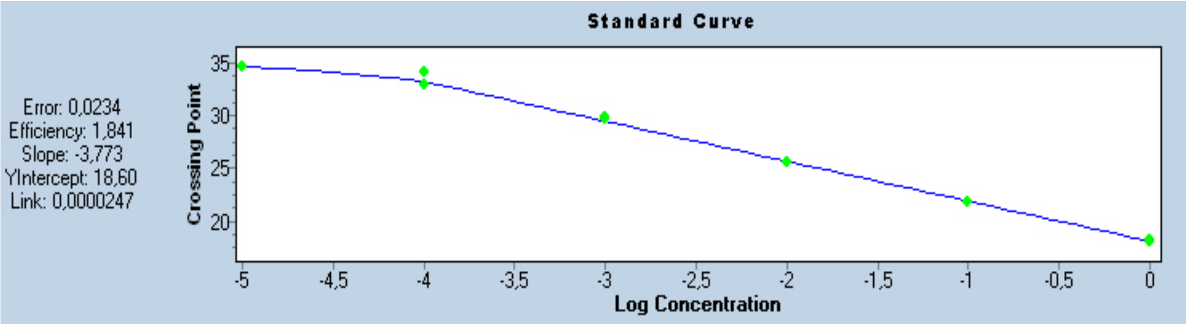

Standard curve for SARS

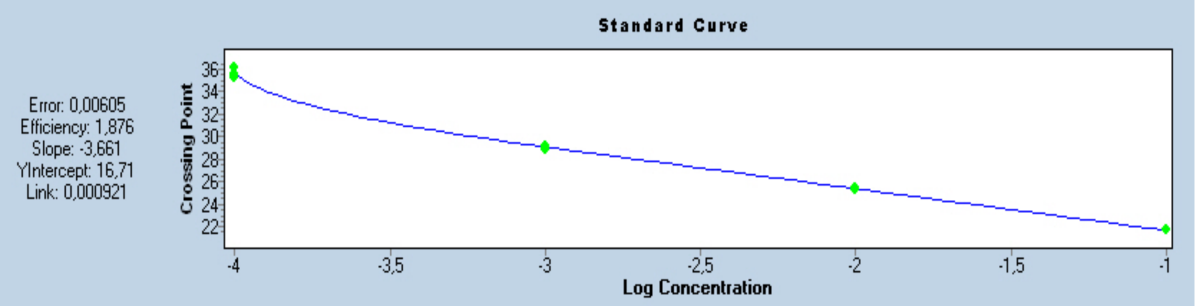

Standard curve for PBGD

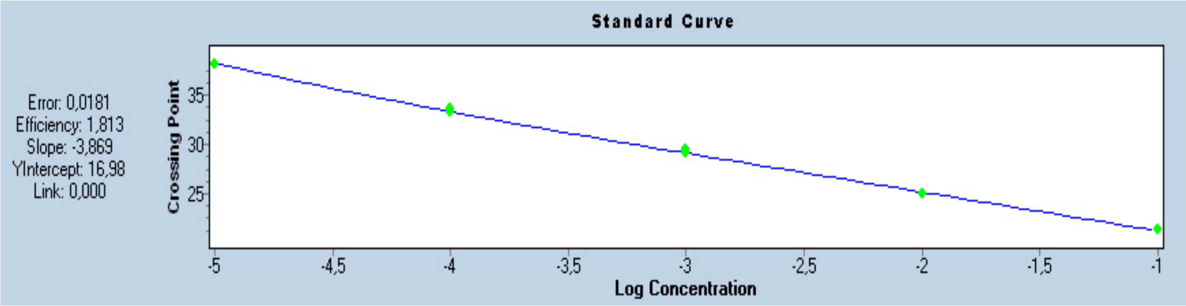

Supplement: Supplementary file 3 [file 362164.f3.pdf]
